# Avian Bornavirus in Free-Ranging Psittacine Birds, Brazil

**DOI:** 10.3201/eid2012.140920

**Published:** 2014-12

**Authors:** Nuri Encinas-Nagel, Dirk Enderlein, Anne Piepenbring, Christiane Herden, Ursula Heffels-Redmann, Paulo A.N. Felippe, Clarice Arns, Hafez M. Hafez, Michael Lierz

**Affiliations:** Freie Universitaet Berlin, Berlin, Germany (N.M. Encinas-Nagel, H.M. Hafez);; Justus Liebig Universitaet, Giessen, Germany (D. Enderlein, A. Piepenbring, U. Heffels-Redmann, C. Herden);; Universidade Estadual de Campinas, São Paulo, Brazil (P.A.N. Felippe, C. Arns)

**Keywords:** Avian bornavirus, free ranging, psittacines, Brazil, proventricular dilatation disease, mononuclear infiltration, birds, parrots, viruses

## Abstract

Avian bornavirus (ABV) has been identified as the cause of proventricular dilatation disease in birds, but the virus is also found in healthy birds. Most studies of ABV have focused on captive birds. We investigated 86 free-ranging psittacine birds in Brazil and found evidence for natural, long-term ABV infection.

Proventricular dilatation disease (PDD) is a fatal disease in birds worldwide, mainly affecting psittacines but also other species (*1*). This disease is characterized by gastrointestinal dysfunction with or without neurologic symptoms. In 2008, two independent research groups identified a novel virus in PDD-affected birds and named it avian bornavirus (ABV) (*2*,*3*); the researchers suggested ABV as the most likely cause of PDD. Subsequent studies have confirmed the association between ABV infection and PDD (*2**–**7*). However, ABV has also been found in healthy birds, indicating that additional factors may be required to cause clinical disease (*4*,*7*). 

To date, 12 genotypes of ABV have been detected from birds in Africa, Europe, North America, Japan, and Australia (*1**–**3*,*6*,*8**–**11*). All recent studies have involved psittacine birds originating from captivity. Villanueva et al. used Western blot assays to test 8 free-ranging psittacine birds from the Peruvian Amazon for the presence of ABV antibodies, but all results were negative (*12*). A literature search yielded no reports of PDD or ABV infection in free-ranging psittacine birds. In this study, we tested apparently healthy, free-ranging psittacine birds captured in Brazil to identify ABV infections or PDD.

## The Study

During December 2009–January 2010, we collected samples from psittacine birds brought to rehabilitation centers (Centro de Rehabilitação de Animais Silvestres [CRAS]) in the federal states of São Paulo (CRAS Parque ecológico Tietê–SP) and Mato Grosso do Sul (CRAS Campo Grande–MS) in Brazil. The birds had been rescued or confiscated from illegal animal trade or holding by environmental police. The 86 birds came from 7 species: *Amazona aestiva* (n = 29), *Aratinga leucophthalmus* (n = 22), *Arara ararauna* (n = 14), *Brotogeris tirica* (n = 10), *Amazona amazonica* (n = 8), *Amazona xanthops* (n = 2), and *Brotogeris chiriri* (n = 1). 

The 49 birds from CRAS do Parque ecológico do Tietê–SP were euthanized for humanitarian reasons because of severe lesions that impeded reintroduction into the wild. The 37 birds from CRAS Campo Grande–MS were in good condition and were submitted for sampling purposes; however, 1 bird died suddenly. These birds had no signs of digestive or neurologic diseases and were anesthetized with inhalational isoflurane before organ sampling or euthanasia.

From dead birds (n = 50), we collected tissue samples from brain, eye, crop, proventriculus, ventriculus, adrenal gland, and heart. From live birds (n = 36), we collected crop biopsy samples from 30. Blood samples (n = 77) and tracheal (n = 78) and cloacal swab (n = 83) specimens were also collected. The collected tissue samples were rapidly embedded in RNALater RNA Stabilizations Reagent (QIAGEN, Hilden, Germany) for further molecular analysis. Real-time reverse transcription PCR (rRT-PCR) was used to detect ABV RNA from cloacal swab and crop biopsy specimens from live birds and from brain or proventriculus samples from dead birds (*3*). Cycle threshold (C_t_) values >36 were considered negative. Genotyping was done according to the method described by Kistler et al. (*2*). Indirect immunofluorescence assay (IIFA) was performed to detect antibodies against ABV in serum samples (*13*). For histopathologic evaluation, collected tissue samples were fixed in 10% buffered formalin. Samples from brain, proventriculus, and ventriculus were embedded in paraffin wax, sectioned at 4 µm, and stained with hematoxylin and eosin. The primary criteria used to diagnose PDD histologically were perivascular or periganglionar mononuclear infiltrates in crop, proventriculus, or both. 

We detected ABV RNA in tissue samples or cloacal swab specimens from 26 (30.2%) sampled psittacine birds; all were identified as type ABV-4. Of these birds, 12 originated from CRAS Tietê and 14 from CRAS Campo Grande. Most (84.6%) of the infected birds were adults, but nestlings (15.4%) were also affected. Antibodies against ABV were found in 19 psittacine birds, but for 15 of them, titers were low (1:10–1:20) and thus the birds were classified as probably, but not clearly, positive. The remaining 4 birds had titers >1:40 and were classified as ABV-antibody positive. 

By evaluating hematoxylin and eosin–stained sections from the crop, brain, and proventriculus of 50 birds and crop biopsy specimens from 30 birds, we determined that 11 (14%) birds had mononuclear infiltrates in the ganglia of the proventriculus or crop, which were diagnosed as suspected PDD (Figure). Seven of these birds also had ABV RNA in tissue samples or cloacal swab specimens or antibodies against ABV in blood samples.

**Figure:**
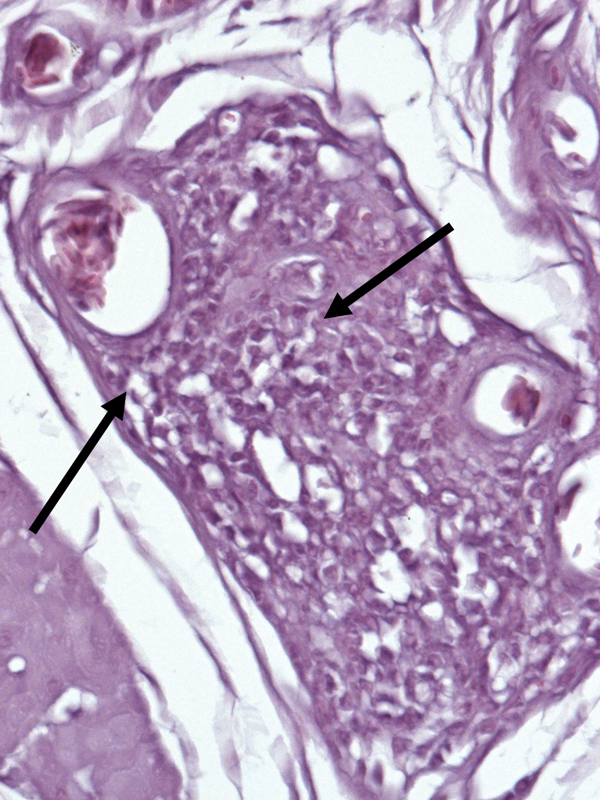
Section of the crop of a white-eyed conure (*Aratinga leucophthalmus*; bird no. 70 [Table 1]) from Brazil that was infected with avian bornavirus genotype 4. Stain shows mononuclear infiltration typical of proventricular dilatation disease (arrows). Hematoxylin and eosin stain; original magnification ×1,000.

## Conclusions

Brazil is known for its variety in free-ranging psittacine birds and is the origin country of endangered species such as the critically endangered Spix’s macaw (*Cyanopsitta spixii*) and the Lear’s macaw (*Anodorhychus leari)*. The spread of infectious pathogens such as ABV is a constant threat for the survival of these species in the wild and to species conservation projects. Hence, identification of disease agents and prevention of infection is critical. 

In this surveillance study, we investigated 86 birds of the family *Psittacidae* originating from the wild from 2 regions of Brazil and found that 40 had signs of ABV infection, PDD, or both (Tables 1, 2). Four birds of 2 species (1 *B. tirica*, bird no. 12; and 3 *A. leucophthalmus*, birds no. 69, 70, and 71) had ABV antibodies in blood and ABV RNA in tissue samples (n = 2), cloacal swab specimens (n = 1), or both (n = 1). Other authors have reported ABV RNA and antibodies against ABV in birds without signs of disease (*4*,*5*,*12*,*14*), which suggests a persistent (*5**,**14*) or a subclinical (*4*,*5*,*9*) course of infection for an indefinite period (*9*,*14*). In our study, 50% of the ABV RNA–positive birds did not show antibodies against ABV by IIFA. Similar findings have also been observed by other authors (*4*,*5*,*14*), which suggests the infection might be at an early stage (*5*,*12*,*13*) or that the virus may escape the immune system (*5*,*15*). Because ABV is unstable in the environment or in fecal material, the virus does not seem to be highly contagious (*14*). Sampling for this study was conducted only once, and because ABV shedding in urofeces is intermittent (*9*), it is possible that some of the tested birds were undetected carriers of ABV and that the prevalence of ABV infection is even wider than supposed. 

**Table 1:** Results of testing of serum and tissue samples from 40 free-ranging psittacine birds that showed signs of ABV infection or PDD disease, Brazil, December 2009–January 2010*

Bird no.	Species	CRAS admission date	Sampling date†	ABV RNA in tissue samples‡	ABV antibody titer in serum sample§	ABV RNA in cloacal swab specimens	Histopathologicsigns of PDD¶
1	*Aratinga leucophthalmus*	2008 Dec 9	2009 Dec 17	–	1:10	–	Cr
2	*Brotogeris tirica*	2008 Oct 14	2009 Dec 17	–	1:10	–	–
4	*A. leucophthalmus*	2008 Aug 2	2009 Dec 17	–	–	–	Cr, Pr
5	*B. tirica*	2009 Oct 22	2009 Dec 17	–	1:20	–	–
6	*B. tirica*	2008 Aug 8	2009 Dec 17	–	–	–	Cr, Pr
8	*B. tirica*	2009 Nov 6	2009 Dec 17	–	1:20	–	–
12	*B. tirica*	2009 Oct 21	2009 Dec 17	33.72	1:160	–	–
18	*Amazona aestiva*	2009 May 15	2009 Dec 17	32.29	–	–	–
19	*A. aestiva*	2009 Jan 15	2009 Dec 17	38.38	–	–	–
16	*Amazona amazonica*	2009 Aug 20	2009 Dec 17	32.50	–	–	–
20	*A. aestiva*	2009 Aug 18	2009 Dec 17	31.22	1:20	–	–
21	*A. aestiva*	2009 Aug 13	2009 Dec 17	34.34	1:20	–	Cr, Br
14	*Brotogeris chiriri*	2009 Mar 6	2009 Dec 17	32.58	–	–	–
17	*A. aestiva*	2008 Dec 12	2009 Dec 17	32.25	–	–	Br
15	*Ara ararauna*	2008 Jun 17	2009 Dec 17	31.44	–	–	Pr
28	*A. aestiva*	2008 Jul 4	2010 Jan 19	33.77	–	–	–
29	*A. aestiva*	2008 Jun 17	2010 Jan 19	–	1:20	–	–
26	*A. amazonica*	2008 Jan 11	2010 Jan 19	33.49	–	–	–
50	*Aratinga leucophthalmus*	2010 Jan 12	2010 Feb 1	35.00	–	–	–
55	*A. leucophthalmus*	2010 Jan 25	2010 Feb 1	–	–	28.85	–
61	*A. leucophthalmus*	2010 Jan 25	2010 Feb 1	–	ND	ND	Pr
67	*Amazona xanthops*	2009 Sep 10	2010 Feb 2	–	–	34.16	–
68	*A. leucophthalmus*	2008 Sep 26	2010 Feb 3	–	1:10	–	–
69	*A. leucophthalmus*	2008 Aug 28	2010 Feb 3	33.44	1:160	–	–
70	*A. leucophthalmus*	2009 Nov 19	2010 Feb 3	34.00	1:160	32.72	Cr
71	*A. leucophthalmus*	2009 Feb 17	2010 Feb 3	–	1:40	34.99	–
73	*A. leucophthalmus*	2008 Mar 19	2010 Feb 3	–	–	34.55	–
75	*A. amazonica*	2009 Jun 19	2010 Feb 3	35.71	–	–	–
76	*A. leucophthalmus*	2010 Feb 2	2010 Feb 3	–	ND	34.17	–
78	*A. aestiva*	2009 Nov 17	2010 Feb 3	–	–	34.01	Cr
79	*A. aestiva*	2009 Jul 8	2010 Feb 3	–	–	34.16	–
80	*A. amazonica*	2008 Dec 5	2010 Feb 3	35.13	1:20	34.38	–
82	*A. aestiva*	NA	2010 Feb 3	–	–	–	Pr
44	*Ara ararauna*	2007 Aug 15	2010 Jan 19	–	1:10	–	–
47	*A. ararauna*	2008 Jun 17	2010 Jan 19	35.11	–	–	Cr, Br
37	*A. aestiva*	2008 Oct 23	2010 Jan 19	–	1:10	–	–
35	*A. aestiva*	2008 Aug 13	2010 Jan 19	–	1:10	–	–
83	*A. aestiva*	2009 Jun 19	2010 Feb 3	ND	1:10	–	ND
85	*A. ararauna*	2009 Oct 29	2010 Feb 4	ND	1:20	32.90	ND
86	*A. ararauna*	2010 Jan 4	2010 Feb 4	ND	1:10	34.59	ND

*–, negative; ABV, avian bornavirus; Br, brain; Cr, crop; CRAS, Centro de Rehabilitação de Animais Silvestres; PDD, proventricular dilatation disease; Pr, proventriculus; NA, not available; ND, not done. †Birds sampled in December and January were from CRAS São Paulo, and birds sampled in February were from CRAS Mato Grosso do Sul. ‡If available, brain samples were used; otherwise crop samples or biopsy samples were tested. RNA from ABV detected by real-time reverse-transcription PCR. Cycle threshold values <36 were considered positive. §Antibodies against ABV in serum sample, detected by indirect immunofluorescence assay. Titers >1:20 were considered positive; titers 1:10–1:20 were considered probably positive. ¶Hematoxylin and eosin staining was used to test all available organ samples from crop, brain, and proventriculus or crop biopsy samples. PDD was diagnosed in birds with ABV RNA, antibodies against ABV, or both and mononuclear infiltrates in or near the ganglia of the crop or proventriculus.

**Table 2:** Detection of ABV RNA, antibodies against ABV, and PDD lesions among free-ranging psittacine birds, by species, Brazil, December 2009–January 2010*

Species	No. birds				
	Total	With ABV RNA in organ sample†	With ABV antibodies in serum sample	With ABV RNA in cloacal swab specimen†	With suspected PDD lesions‡
*Amazona aestiva*	29	6	0	2	4
*Aratinga leucophthalmus*	22	3	3	5	4
*Ara ararauna*	14	2	0	2	2
*Brotogeris tirica*	10	1	1	0	1
*Amazona amazonica*	8	4	0	1	0
*Amazona xanthops*	2	0	0	1	0
*Brotogeris chiriri*	1	1	0	0	0
Total	86	17	4	11	11

*ABV, avian bornavirus; PDD, proventricular dilatation disease. †By real-time reverse transcription PCR. ‡By hematoxylin and eosin stain.

ABV RNA in cloacal swab specimens reinforces the suggestion of a gastrointestinal/urogenital location for the virus in the free-ranging psittacine bird population in these areas of Brazil. Unless infection during captivity in the rehabilitation centers can be proven, infection in the wild must be considered. We found ABV RNA by rRT-PCR in a white-eyed conure nestling (*Aratinga leucophthalmus*; bird no. 76) that was introduced into the CRAS Mato Grosso do Sul rehabilitation center 1 day before sampling. Likewise, 6 birds of 3 species (*B. tirica*, birds no. 5, 8, and 12; *A. leucophthalmus*, birds no. 50 and 55; and *A. ararauna*, bird no. 86) that arrived in the centers 2 months to 1 week before sampling showed either antibodies against ABV or ABV RNA (Table 1). 

In summary, our findings indicate the apparent existence of ABV infection in the free-ranging psittacine bird population in 2 areas of Brazil. The presence of antibodies in the birds we tested may indicate long-term infection in which ABV viral shedding and transmission are not unusual. Our findings provide evidence of natural infection with ABV in free-ranging psittacine birds, the development of histopathologic lesions typical of PDD, and the occurrence of this disease in the natural habitat of these birds in South America.
